# ProDesign Logic Files Effect on Apical Foramen Wear and Shape Transformation After Foraminal Enlargement

**DOI:** 10.3390/dj12100330

**Published:** 2024-10-16

**Authors:** Miguel Christian Castillo Marin, Amjad Abu Hasna, Marcos Frozoni, Mariana Gadelho Gimenez Diamantino, Caroline Trefiglio Rocha, Marcia Carneiro Valera, Cláudio Antonio Talge Carvalho

**Affiliations:** 1 Department of Restorative Dentistry, Endodontics Division, Institute of Science and Technology, São Paulo State University (ICT-UNESP), São José dos Campos 12245-000, SP, Brazil; miguel.endodontia@gmail.com (M.C.C.M.); mariana.gimenez@unesp.br (M.G.G.D.); caroline.trefiglio@unesp.br (C.T.R.); marcia.valera@unesp.br (M.C.V.); claudio.talge@unesp.br (C.A.T.C.); 2 School of Dentistry, Universidad Espíritu Santo, Samborondón 092301, Ecuador; 3 Department of Endodontics, São Leopoldo Mandic School of Dentistry, Campinas 13045-755, SP, Brazil; marcosfrozoni@gmail.com

**Keywords:** apical foramen, root canal preparation, foraminal enlargement

## Abstract

Background/Objectives: This study aimed to evaluate the effect of ProDesign Logic files 45.01 and 50.01 on apical foramen wear and shape transformation after foraminal enlargement at tooth length (TL) and 1 mm beyond. Methods: Sixty freshly extracted single-rooted lower human premolars teeth were distributed randomly into five groups (*n* = 12): Group 1 (40.05 WL) (control): No foraminal enlargement, Group 2 (45.01 TL): Specimens underwent foraminal enlargement with 45.01 Prodesign Logic file at TL; Group 3 (45.01 TL + 1): Specimens underwent foraminal enlargement with 45.01 Prodesign Logic file at 1 mm beyond TL; Group 4 (50.01 TL): Specimens underwent foraminal enlargement with 50.01 Prodesign Logic file at TL; Group 5 (50.01 TL + 1): Specimens underwent foraminal enlargement with 50.01 Prodesign Logic file at 1 mm beyond TL. Scanning electron microscopy was used to evaluate apical foramen variations and shape alterations. Data were analyzed by Student’s *t*-test, Kruskal–Wallis, and Mann–Whitney test. Results: Significant differences (*p* < 0.01) were observed among all tested groups compared to the control group in terms of the average worn area of the apical foramen. Foraminal enlargement led to significant shape transformation, resulting in round-shaped foramina. Conclusion: ProDesign Logic 45.01 and 50.01 files at TL causes notable wear and round-shaped foramina.

## 1. Introduction

Cleaning and shaping of the root canal system are considered the main concepts to obtain successful endodontic treatment [1]. This process involves eliminating microorganisms, their byproducts, and contaminated tissues [2,3] using different chemical substances, such as endodontic irrigants and intracanal medications [4,5], along with mechanical instruments [6] throughout the entire root canal system. More specifically, the apical third of the root canal is considered a critical area because of its anatomical variations, limited access, and variety of persistent microorganisms, which are often involved in treatment failures [7,8,9].

The instrumentation and obturation limits of the root canal are indicated in diverse studies to extend up to the apical constriction, or cementodentinal junction (CDJ) [10]. However, two concepts should be considered regarding the cleaning of the apical foramen. The first is apical patency, which involves passively passing a small-diameter instrument through the foramen without enlarging it to keep it accessible during the instrumentation [11]. The second is the apical foraminal enlargement, which involves using a higher diameter instrument to enlarge the apical foramen to effectively disinfect the region [12].

The apical foraminal enlargement is a controversial topic in endodontics [13], while some studies highlight its benefit in more decontamination, particularly in necrosed pulp cases [14] and reduction in inflammatory markers [15], resulting in substantial success rates ranging between 81.1 and 87.4% [16], concerns remain about the risks associated with over-instrumentation. These risks include periapical tissue damage, postoperative pain because of debris extrusion, root weakening, perforation, and difficulty in achieving a proper seal during obturation [13,17,18]. Still, many studies have been developed with the purpose of analyzing the reduction of periapical biofilm, cleaning of root canals, and postoperative symptoms associated with foraminal enlargement after endodontic treatment in which there are conflicting results regarding the size and taper of the apical preparation [19,20,21].

In this context, two additional principles [1] are to be considered while evaluating the effect of the foraminal enlargement on the outcome of the root canal treatment. The first is the quantity of the worn tissue and how this affects the apical foramen size, and the second is the apical foramen transportation, which should be avoided.

ProDesign Logic (Easy Equipamentos Odontológicos, Belo Horizonte, MG, Brazil) is a rotary nickel–titanium instrument made of controlled memory thermal treated wire, according to the manufacturer, it has a double helix cross section for tapers 0.03, 0.05, and 0.06; a triple helix for taper 0.04; and a quadruple helix for taper 0.01. It offers a variety of tapers designed to minimize procedural errors. Smaller tapers particularly are indicated for periapical enlargement as, according to the manufacturer, it reduces the impact on the foramen wear and the apical transformation possibility [22].

With a hypothesis that using ProDesign Logic could have no impact on apical foramen wear and shape transformation. This study aimed to evaluate the effect of ProDesign Logic files 45.01 and 50.01 on apical foramen wear and shape transformation after foraminal enlargement at tooth length and 1 mm beyond.

## 2. Materials and Methods

### 2.1. Sample Size Calculation and Tooth Selection

This study was conducted after approval of the Ethics Committee for Research Involving Human Beings of the Institute of Science and Technology of São Paulo State University (protocol 6.879.354). All donor patients signed a free and informed consent form. The specimens were collected from patients undergoing tooth extraction who had given consent for their use in this study. The present study was conducted in compliance with the Helsinki Declaration for research on human subjects.

The sample size was calculated based on population parameters, sampling error (8%), confidence level (90%), and population distribution (homogeneous 80/20). The comparison of mean values from five groups in the study was obtained from a pilot study, which was performed to assess the feasibility of foraminal enlargement with varying file sizes, guiding the final grouping for the main study. Then, power tests were conducted for the ANOVA (1-way) model using the Minitab program (version 17.1, Microsoft, Redmond, WA, USA) for the variable area. It was found that with a sample size of 12 and variability (standard deviation) of 12,000 pixels, it is possible to detect a difference of 2000 pixels with a test power above 80%.

Sixty freshly extracted single-rooted lower human premolar teeth were included in this study. These teeth were immersed in 2.5% sodium hypochlorite solution “NaOCl” for 6 h for decontamination and promoting the dissolution of tissue residues adhered to the dental surfaces. Then, teeth were rinsed and brushed under running water, and finally, they were dried and preserved in saline solution (Eurofarma, São Paulo, SP, Brazil) to prevent dehydration until they were ready for use.

### 2.2. Inclusion, Exclusion, and Non-Inclusion Criteria

As inclusion criteria, only lower premolars with (I) single fully formed root canal, (II) closed apex, (III) caries-free, (IV) no calcification in the root canal or pulp stones in the pulp chamber, (V) no previous root canal treatment, (VI) straight or slightly curved roots (25° and 35°) [23]. Conversely, all premolars that did not meet the inclusion criteria were excluded.

As non-inclusion criteria, teeth underwent foramen diameter analysis and standardization under an X20 stereomicroscope (Stemi 2000—Karl Zeiss, Oberkochen, Germany) at 80× magnification, in which only teeth with foraminal diameter in their longest axis of up to 0.40 mm were included, otherwise, were not included.

### 2.3. Specimens’ Preparation and Experimental Groups

The crown of each premolar was cross-cut at the cemento-enamel junction using a carborundum disc (KG Sorensen, Barueri, SP, Brazil) while under refrigeration, resulting in a root length of approximately 16 ± 0.5 mm. Subsequently, the root canals were explored using a K-file #10 (Dentsply Maillefer, Ballaigues, Switzerland). The working length was standardized at 15 mm. Then, all specimens were instrumented with 25.06 and 40.05 Prodesign Logic files (Easy Equipamentos Odontológicos, Belo Horizonte, MG, Brazil) to the working length with continuous rotary movement at speed of 950 rpm and torque of 4.0 Ncm. Then, specimens were distributed randomly into five groups (*n* = 12):

- Group 1 (40.05 WL) (control): No foraminal enlargement, specimens were instrumented only with 25.06 and 40.05 Prodesign Logic files at the apical constriction (AC) limit, which was considered the tooth length minus 1 mm (working length);
- Group 2 (45.01 TL): Specimens underwent foraminal enlargement with 45.01 Prodesign Logic file at the tooth length (TL);
- Group 3 (45.01 TL + 1): Specimens underwent foraminal enlargement with 45.01 Prodesign Logic file at 1 mm beyond the tooth length (TL);
- Group 4 (50.01 TL): Specimens underwent foraminal enlargement with 50.01 Prodesign Logic file at the tooth length (TL);
- Group 5 (50.01 TL + 1): Specimens underwent foraminal enlargement with 50.01 Prodesign Logic file at 1 mm beyond the tooth length (TL).

The specimens were irrigated with 5 mL of 2.5% NaOCl at each instrument change. They were then filled with 17% Ethylenediaminetetraacetic acid (EDTA) solution (Fórmula e Ação, São Paulo, São Paulo, Brazil) for 3 min, with agitation using a K-file #20 file until 1 mm short of the working length. Finally, the canals of the specimens were rinsed with 5 mL of saline solution to remove any potential residues from the chemical solutions used. Subsequently, the canals were dried with paper points.

### 2.4. Scanning Electron Microscopy

The specimens were fixed in a 2.5% glutaraldehyde solution for 2 h and dehydrated using solutions of increasing alcohol concentration (10%, 25%, 50%, 70%, 90%, and 100%). Then, they were kept at 37 ± 1 °C for 24 h [4]. Next, the specimens were fixed, using chemically activated acrylic resin, on a small peripheral collar made of silicone with a diameter of 3 mm, and this collar was fixed onto standardized stubs. The silicone collar was used to create a locking system for the specimens, preventing rotation within the stub. This entire process created customized specimens that fit perfectly onto the stubs, maintaining the same position when placed into the scanning electron microscopy (SEM).

Subsequently, specimens’ metallization was performed in a vacuum chamber (Desk II—Denton Vacuum—Buffalo, NJ, USA) with a 120 s sputtering (gold plating) period before being taken to the SEM. The images were obtained in two stages.

The first images were taken without any instrumentation or exploration of the root canal to record the initial morphology of the apical foramen (Baseline images). Coordinates (*x*, *y*, and *z* axes), angles, and positioning distances of the specimens on the SEM stage were recorded to ensure similar images when repositioned under the microscope. For the second stage, after instrumentation of the teeth, the previous procedures were repeated to obtain images with SEM. The instrumented teeth were distributed onto their respective stubs, positioned in the same initial position, and specimens were locked into the locking button. When visualizing the apical foramen in the SEM, the same angles and distances recorded during the capture of initial images prior to chemical–mechanical preparation were respected. Consequently, two identical photomicrographs of each sample were obtained using scanning electron microscopy (JEOL—Model JSM T330A—Tokyo, Japan) at a standard magnification of 100×.

### 2.5. Evaluation of Apical Foramen Variations

The baseline images taken before instrumentation of the specimens were analyzed using the Image J 1.52 K analysis program (National Institutes of Health, Bethesda, Rockville, MD, USA). The area of the foramen was measured using the “freehand selections” tool, manually delineating the area before and after instrumentation. To ensure consistency, all measurements were performed by a single operator trained and calibrated in the use of Image J software. A calibration test was conducted, yielding a coefficient of intra-examiner reliability of 93%.

### 2.6. Evaluating Foraminal-Shape Alteration

The regularity of the apical foramina was calculated using Pearson’s coefficient of variation (CV). This coefficient represents the ratio between the standard deviation (Sd) and the mean (M) of a set of measurements (CV = Sd/M). In the images of the apical foramina, analyzed at 100× magnification, eight radial measurements were taken from the center of the largest diameter to the edge of the apical foramen. To guide these eight measurements, a template consisting of eight lines forming a 45° angle between them, distributed from a central point, was created. The images of the apical foramen before and after instrumentation, along with the eight-ray template, were superimposed using the IrfanView Version 4.53 editing program (Copyright © 1996–2019 by Irfan Skiljan). The overlaid images were saved in jpeg format and then taken for distance measurement using the Image J 1.52 K program (National Institutes of Health, USA). Measurements were taken between the center of the template and the points where the eight lines intersected the edge of the analyzed apical foramen. The chosen unit of measurement was pixels. For each of the 60 specimens, the CV of the original foramen (before treatment) and the foramen after instrumentation were determined. The formula used to calculate the CV was: CV = {sd/[(r1 + r2 + r3 + r4 + r5 + r6 + r7 + r8)/8]} × 100. Therefore, CV represents the standard deviation divided by the average of the measurements of the 8 radii in each evaluated apical foramen, multiplied by 100. In practice, the lower the CV value, the closer one would expect the apical foramen to be to a circle. Conversely, a higher CV value would suggest a more oval or irregular shape of the apical foramen.

### 2.7. Statistical Analysis

- Normality tests (Shapiro–Wilk and Kolmogorov–Smirnov test) were used to analyze the data.
- Paired *t*-test was used to analyze the variation in the foramen before and after instrumentation within the same group. The same test was applied in comparing the Coefficients of variation (CV) before and after instrumentation for foraminal shape transformation analysis.
- Kruskal–Wallis test to compare the variation in foraminal area before and after instrumentation among the different groups, followed by Dunn–Bonferroni test as the post hoc test.

## 3. Results

**Figure 1:**
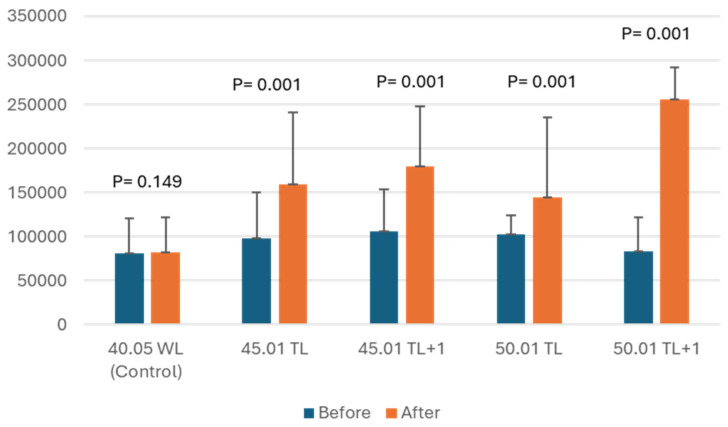
Mean and standard deviation (SD) of apical foramen area measurement (in pixels) before and after the treatments.

There was a statistically significant difference in the increase in the worn area of the apical foramen, resulting in a significant apical foramen enlargement in all the tested groups except of the control group, as shown in Figure 1.

**Figure 2:**
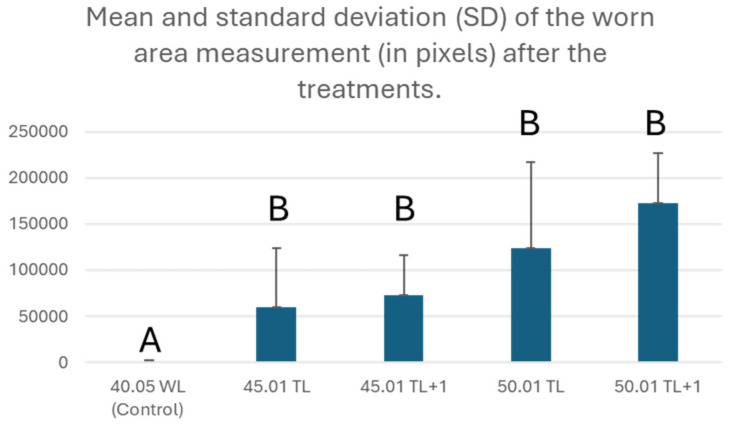
Mean and standard deviation (SD) of the worn area measurement (in pixels) after the treatments. Different uppercase letters (A, and B) indicate statistically significant differences among the groups.

When we compared the average worn area of the apical foramen between the groups (Figure 2), it was observed that there was a statistically significant difference (*p* < 0.01) among all the tested groups when compared to the control group.

**Figure 3:**
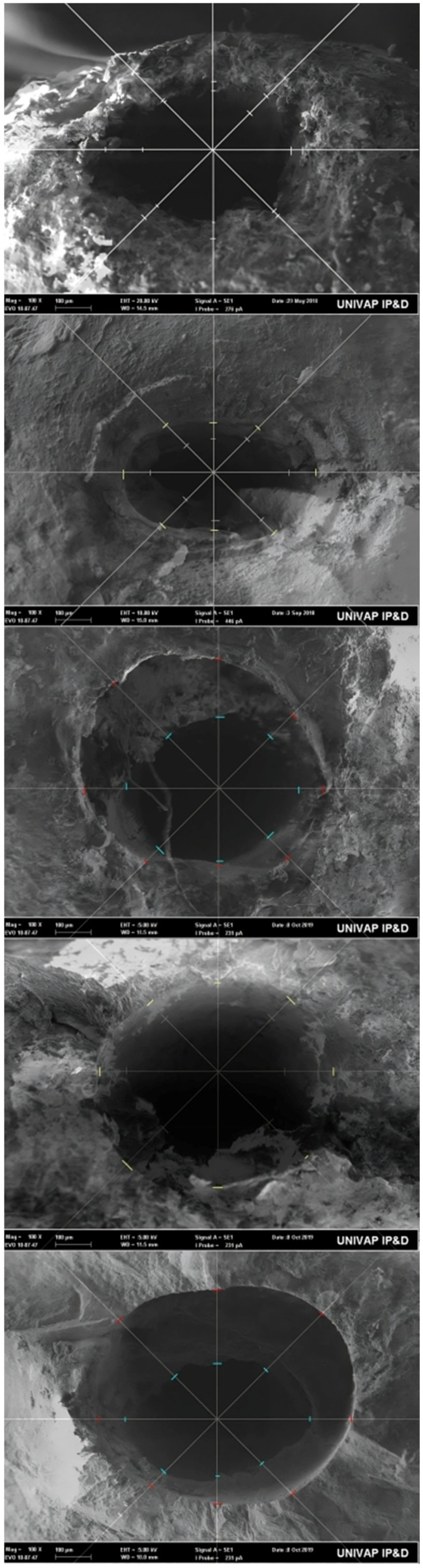
Overlay of SEM images of the apical foramen before and after instrumentation in the different groups. From the first to the last, the groups: 40.05 WL, 45.01 TL, 45.01 TL + 1, 50.01 TL, and 50.01 TL + 1.

Figure 3 represents the overlay of the SEM images of the apical foramen before and after instrumentation in the different groups. In these images, we can descriptively observe the degree of foramen shape transformation, enlargement, and regularization following foraminal enlargement.

**Figure 4:**
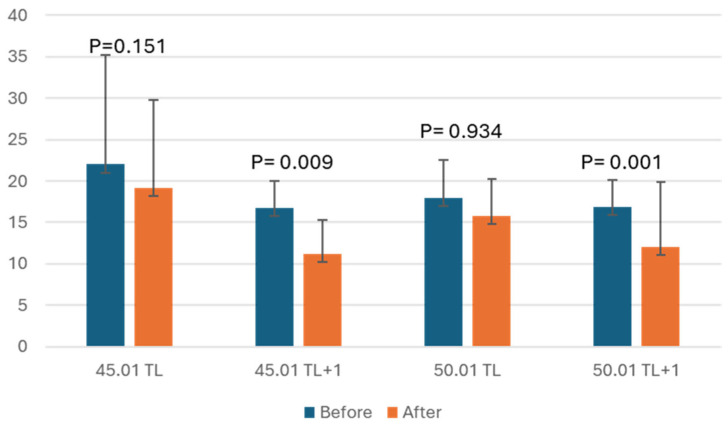
Mean and standard deviation (SD) in pixels and variance analysis of the coefficient of variation of the apical foramen before and after treatments.

The mean values and standard deviation of the coefficients of variation are found in Figure 4, besides the results of the statistical analysis (paired *t*-test) comparing before and after instrumentation in the different groups, in which group 45.01 TL + 1 had the lowest average coefficient of variation, while the highest value was observed in group 45.01 TL.

We can note that there was a reduction in the average coefficient of variation after instrumentation in all experimental groups; however, this difference was statistically significant only in the groups 45.01 TL + 1 and 50.01 TL + 1, both instrumented at 1 mm beyond the length limit of the tooth. This significance was not observed in the groups 40.01 TL and 50.01 TL, whose foraminal enlargement was at tooth length.

When comparing the groups 45.01 TL and 50.01 TL, both instrumented at tooth length, we noticed that the group with greater apical enlargement resulted in a lower average CV value. When instrumented 1 mm beyond the length of the tooth, group 45.01 TL + 1 presented a lower average CV value compared to the larger caliber group 50.01 TL + 1.

Considering foraminal enlargement with instruments of the same caliber, group 45.01 TL + 1 achieved a better result with an average CV of 11.23% compared to the 19.17% presented by group 45.01 TL, instrumented at the length limit of the tooth.

Based on the results, comparing group 50.01 TL with group 50.01 TL + 1, we observe that instrumentation 1 mm beyond the length of the tooth had a lower average CV value (12.05%) than the group with foraminal enlargement at the tooth length limit (15.83%).

## 4. Discussion

The apical third often exhibits multiple branches of the main canal [24]. Alongside anatomical variability, the foramen and its branches within the apical third represent crucial pathways connecting the main canal to the periodontal ligament space. These pathways can facilitate the transportation of bacteria responsible for periapical diseases, rendering the apical region intricate and demanding [25]. The aim of this study was to evaluate the effect of ProDesign Logic files 45.01 and 50.01 on apical foramen wear and shape transformation after foraminal enlargement at tooth length and 1 mm beyond.

In this study, the chosen teeth underwent prior analysis regarding the morphology of their foramens using a stereoscopic magnifying glass. This pre-screening aimed to standardize foraminal size by excluding those with diameters smaller than 0.30 to 0.40 mm (equivalent to a #40 file), pronounced curvatures, and apical lacerations. This selection process enhanced uniformity among specimens, minimizing variables that could affect biomechanical preparation with mechanical instruments.

Another point to be emphasized is that the developed methodology for this study was the inclusion of the coronary portion of the roots in the metallic stubs in a customized manner, preventing the rotation of the specimens and positioning them on the SEM mobile platform in a way that allowed them to be evaluated always in the same position. Nevertheless, the photomicrographs showed slight differences in positioning that, due to the magnification used, were considered negligible. Thus, it was possible, even using the scanning electron microscope, to produce comparable images, repeated with each treatment performed.

The use of SEM in this study was chosen for its ability to provide high-resolution, detailed images of the root canal’s surface morphology, including the apical foramen. SEM allows for a close examination of microstructural changes, such as tissue wear and material deposition [26], which are crucial for evaluating the effects of instrumentation on the root canal. Conversely, other methods like Cone Beam Computed Tomography and micro-CT are valuable for providing three-dimensional imaging and assessing root canal anatomy and curvature [27]. Still, SEM was specifically selected in this study due to its superior ability to analyze surface topography at a microscopic level.

It was found in the present study that there was a statistically significant difference in the increase in the worn area of the apical foramen, resulting in a significant apical foramen enlargement in all the tested groups except the control group, which had no foraminal enlargement. Therefore, the hypothesis of this study of ProDesign Logic could have no impact on apical foramen wear should be rejected. These results are in agreement with the outcomes of the recent micro-computed tomography study that concluded that instrumentation at tooth length, or 1 mm beyond, resulted in foraminal enlargement, even using relatively lower diameter instruments (20, 25, and 30) [27]. The systematic review by Campos et al. (2024) concluded that both in vivo and ex vivo studies suggest that foramen enlargement enhances tissue repair and reduces bacterial load within the root canal [20].

In addition, in this study, we can note that there was a reduction in the average coefficient of variation after instrumentation in all experimental groups; however, this difference was statistically significant only in the groups 45.01 TL + 1 and 50.01 TL + 1, both instrumented at 1 mm beyond the length limit of the tooth, which means that the foramina were found in a round shape; however, this significance was not observed in the groups 40.01 TL and 50.01 TL whose foraminal enlargement was at tooth length suggesting a more oval or irregular shape of the apical foramen. In another study [28], it was found that the apical enlargement of up to 1.5 mm makes the apical foramen more oval, agreeing with the results of our study. This deformation of the foramen shape is affected by the file size, motion, and design and by the enlargement limit [13].

It is worth highlighting here that round-shaped foramina are generally associated with more predictable and uniform obturation due to the symmetry of the canal, which allows for a better seal and reduces the risk of voids or gaps. This uniformity is advantageous in achieving a hermetic seal, which is crucial for preventing reinfection and ensuring long-term treatment success. On the other hand, oval-shaped foramina present a greater challenge during obturation due to their irregular geometry [29,30]. One more point to be emphasized is the enlargement limit. When comparing the groups 45.01 TL and 50.01 TL, both instrumented at tooth length, we notice that the group with greater apical enlargement resulted in a lower average CV value, which means a round-shaped foramen. However, when instrumented 1 mm beyond the length of the tooth, group 45.01 TL + 1 presented a lower average CV value compared to the larger caliber group 50.01 TL + 1 (*p* < 0.01). This means clinically that foraminal enlargement up to the tooth length is more indicated, according to the outcomes of the present study, as it will result in a round-shaped foramen, which facilitates the instrumentation and obturation of the root canal [31] more than oval-shaped foramen [32]. Larger taper instrumentation has been shown in other studies to cause deviation from the original foramen anatomy [33], where deformation levels were observed [34].

Overall, the results showed that the enlargement significantly regularized the apical foramen. However, foramen enlargement increases the possibility of endodontic cement extrusion, which may delay periapical healing [33]. However, it should be performed with less intensity or with other instruments and/or techniques in cases where a pronounced apical curvature of the canal is observed. Further research is needed to refine the techniques and instruments used for apical enlargement in curved canals to mitigate these risks.

This study, being in vitro, carries inherent limitations related to the laboratory setting, which may not fully replicate clinical conditions. For example, the absence of natural periodontal ligament support in extracted teeth may influence the behavior of the root canal during instrumentation. Moreover, while this study provides valuable insights into foraminal enlargement, the clinical impact of these findings, particularly in necrotic cases or cases with more complex anatomies, requires further investigation through in vivo studies.

Nevertheless, our findings suggest that using instruments with a low taper, such as 0.01, can facilitate apical enlargement and improve the overall shape regularity of the foramen, particularly in necrotic cases. Future research should explore the impact of different instrument designs and motion techniques on apical enlargement outcomes, especially in cases with significant anatomical variations.

## 5. Conclusions

The foraminal enlargement with ProDesign Logic 45.01 and 50.01 at tooth length results in a significant worn area and leads to significant foramen regularization, producing round-shaped foramina This effect is particularly when extending instrumentation 1 mm beyond the tooth length. However, care should be taken in cases with pronounced apical curvature, as further research is needed to optimize techniques in such scenarios.

## Data Availability

The raw data supporting the conclusions of this article will be made available by the authors upon request.
